# Epigenetics: Linking Early Postnatal Nutrition to Obesity Programming?

**DOI:** 10.3390/nu11122966

**Published:** 2019-12-05

**Authors:** Lucie Marousez, Jean Lesage, Delphine Eberlé

**Affiliations:** University Lille, EA4489 Environnement Périnatal et Santé, Équipe Malnutrition Maternelle et Programmation des Maladies Métaboliques, F-59000 Lille, France

**Keywords:** obesity, early postnatal nutrition, developmental programming, epigenetics, DNA methylation, breast milk

## Abstract

Despite constant research and public policy efforts, the obesity epidemic continues to be a major public health threat, and new approaches are urgently needed. It has been shown that nutrient imbalance in early life, from conception to infancy, influences later obesity risk, suggesting that obesity could result from “developmental programming”. In this review, we evaluate the possibility that early postnatal nutrition programs obesity risk via epigenetic mechanisms, especially DNA methylation, focusing on four main topics: (1) the dynamics of epigenetic processes in key metabolic organs during the early postnatal period; (2) the epigenetic effects of alterations in early postnatal nutrition in animal models or breastfeeding in humans; (3) current limitations and remaining outstanding questions in the field of epigenetic programming; (4) candidate pathways by which early postnatal nutrition could epigenetically program adult body weight set point. A particular focus will be given to the potential roles of breast milk fatty acids, neonatal metabolic and hormonal milieu, and gut microbiota. Understanding the mechanisms by which early postnatal nutrition can promote lifelong metabolic modifications is essential to design adequate recommendations and interventions to “de-program” the obesity epidemic.

## 1. Introduction

Obesity is a major risk factor for many serious chronic pathologies, including type 2 diabetes, cardiovascular diseases, and cancers [1]. The worldwide prevalence of obesity nearly tripled between 1975 and 2016. More dramatically, the prevalence of overweight and obesity among children and adolescents has risen from just 4% in 1975 to just over 18% in 2016 [2]. Despite constant research and public policy efforts, the obesity epidemic continues to be a major public health threat, emphasizing the need to initiate new preventative and therapeutic strategies. It is widely accepted that obesity is mainly determined by insufficient physical activity and excess consumption of energy-dense foods, which ultimately lead to positive energy balance and fat mass accumulation. However, the failure of current strategies aimed at promoting long-term weight loss indicate that individual body weight is not just a question of will power. Recent evidence has indicated that nutrient imbalance in early life, from conception to infancy, influences later obesity risk [3,4], suggesting that obesity could result from “developmental programming”. Interestingly, epidemiologic and animal studies have shown that early postnatal nutrition alone, independently of the in utero milieu, can influence obesity risk. Indeed, breastfeeding, as opposed to formula feeding, is moderately but consistently protective against rapid neonatal weight gain and susceptibility to adult obesity [5,6,7,8,9,10]. Yet, the benefits of breast milk may be modulated by its composition [11,12], highlighting the need to better characterize early infant nutrition and its lifelong consequences. Animal studies have confirmed that altered nutrition in mothers or pups during lactation-suckling is sufficient to determine long-term adiposity in offspring [13,14,15,16]. Understanding the mechanisms by which early postnatal nutrition influences the adult body weight “set point’’ would offer the opportunity to design targeted recommendations and interventions for long-term benefits.

One favored hypothesis to explain the developmental programming of obesity is the potential role of epigenetic processes [17]. Epigenetics refers to the study of heritable changes in gene expression, which result from chromatin modifications (e.g., DNA methylation, histone modifications) without change in DNA sequence. Importantly, epigenetic processes exhibit some degree of plasticity in response to environmental stimuli. As such, epigenetics has been considered a prime mechanism of cellular reprogramming and memory of past environment [18]. In this review, we evaluate the possibility that early postnatal nutrition programs obesity risk via epigenetic mechanisms. First, we outline the nature and dynamics of epigenetic phenomena taking place in key metabolic organs, specifically in the early postnatal window, highlighting that developmental epigenetics extends far beyond the fetal period. In the second part, we review animal models of obesity programming triggered by nutritional alteration in the lactation-suckling period and summarize the evidence of epigenetic remodeling observed in these models. The results from the few human studies investigating the epigenetic effects of breastfeeding are also examined. While the literature continues to document epigenetic alterations in obesity programming, it remains challenging to determine the molecular mechanisms by which postnatal nutrition affects DNA methylation and obesity programming. Examining recent animal studies, we discuss the role of breast milk fatty acids, neonatal metabolic and hormonal milieu, and gut microbiota in the epigenetic programming of obesity. Our long-term goal is to define the precise mediators and pathways by which postnatal nutrition can promote lifelong metabolic (mal)adaptations in order to design potential diagnostic and therapeutic tools that could be used to “de-program” this vicious circle of obesity propagation.

## 2. The Early Postnatal Period: A Critical Developmental Epigenetic Window

### 2.1. DNA Methylation and Demethylation

**Figure 1:**
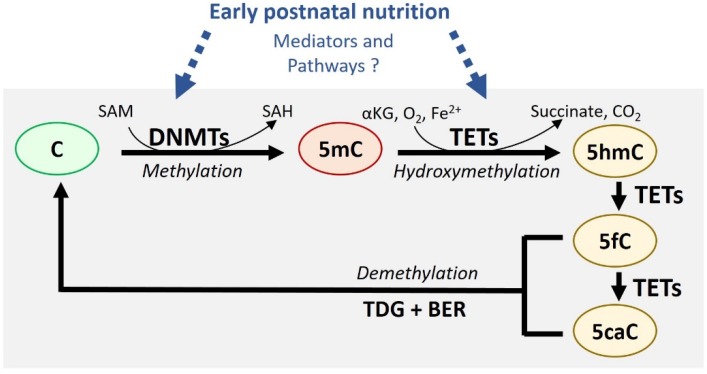
DNA methylation is catalyzed by DNA Methyltransferase (DNMTs) which add a methyl group to the carbon 5 of cytosine (C) in CpG dinucleotides to form 5-methylcytosine (5mC). The methyl donor, S-adenosyl-L-methionine (SAM), is thereby converted to S-adenosyl-L-homocysteine (SAH). DNA methylation typically changes chromatin from an active (green) to a repressive state (red) where gene expression is reduced. 5mC can be oxidized by Ten-Eleven Translocation dioxygenases (TETs) to generate 5hmC (hydroxymethylation) which can be further oxidized in 5-formyl cytosine (5fC) and 5-carboxyl cytosine (5caC). This iron (Fe^2+^)-catalyzed reaction concomitantly converts α-ketoglutarate (αKG) and oxygen (O_2_) to succinate and carbon dioxide (CO_2_). The conversion of 5mC to its oxidized derivatives (5hmC, 5fC and 5caC) is expected to lead to DNA demethylation by active and passive mechanisms. 5fC and 5caC can be excised by Thymine DNA glycosylase (TDG), followed by base excision repair (BER) to generate C. Modifications can be also lost during cell replication in the absence of DNA maintenance methylation by DNMT1. Nutrition is believed to affect DNA methylation/demethylation processes by altering substrates and cofactors necessary for these reactions or by changing expressions and/or activities of DNMTs and TETs enzymes. While the biochemical pathways by which early postnatal nutrition specifically could influence these processes are largely unknown, we discuss mediators and candidate pathways in Section 4.

The first and most studied epigenetic mechanism is DNA methylation. It is defined by the addition of a methyl group on the cytosine of cytosine-guanine dinucleotides (CpG) by DNA methyltransferase (DNMTs) enzymes to form 5-methylcytosine (5mC) [19] (Figure 1). Other epigenetic marks include the post-translational modification of histone proteins, but mechanisms for their inheritance are less well defined. As such, DNA methylation has emerged as the predominant mechanism for long-term cellular memory [20] and is the main focus of this review. De novo DNA methylation is established by DNMT3A and DNMT3B isoforms while DNMT1 copies the DNA methylation pattern during replication and mitosis [18]. DNA methylation in promoters or enhancers is usually associated with gene silencing due to the disruption of transcription factor DNA binding and/or the recruitment of repressor complex(es).

While DNA methylation has long been considered permanent after it has been established, the recent discovery of Ten-Eleven Translocation (TET) methylcytosine dioxygenase enzymes (TET1, TET2, TET3) has revealed a more dynamic picture. TET enzymes convert 5mC to 5-hydroxymethylation (5hmC), as well as further oxidized intermediates (5-formylcytosine (5fC) and 5-carboxycytosine (5caC) that prompt active DNA demethylation [21] (Figure 1). Indeed, these modified cytosines are rapidly excised by thymine DNA glycosylase (TDG), after which they are replaced by unmethylated cytosines through base excision repair (BER) mechanisms. However, 5hmC is readily detected in many cell types in regions near promoters and enhancers where it correlates positively with gene expression [22,23]. This suggests that 5hmC could serve as an epigenetic mark with specific functions. Of note, many studies have mapped the DNA methylation landscape using DNA bisulfite conversion methods which do not distinguish between 5mC and 5hmC [24]. Given the opposing effects of 5mC and 5hmC on gene expression, future studies should increasingly employ available methods that discriminate 5mC from 5hmC [25] to clarify interpretation of the results.

It is well known that nutrition influences the DNA methylation landscape in various tissues and cells. Indeed, a large number of studies have reported broad or locus-specific DNA methylation changes following various dietary interventions during perinatal or adult periods. However, far fewer studies have focused their efforts on understanding the molecular mechanisms by which nutrients drive these epigenetic changes. It is believed that nutrition affects DNA methylation/demethylation processes by altering the substrates and cofactors necessary for these reactions, such as methyl donors, SAM, and SAH, or by changing the expression and/or activity of DNMTs and TETs enzymes [26,27] (Figure 1). It is also plausible that dietary components could have different epigenetic impacts, depending on the time period of exposure. In this context, it is crucial to outline the nature and dynamics of epigenetic phenomena taking place in key metabolic organs, specifically in the early postnatal window.

### 2.2. Postnatal Developmental and Epigenetic Dynamics in Organs of Energy Homeostasis

The potential role of epigenetics in developmental programming has often been addressed in the context of the fetal period in which DNA methylation patterning is known to be particularly active to allow normal tissue development. However, recent evidence has indicated that the early postnatal period is also a developmental window of major interest with important epigenetic plasticity. In mammals, the early postnatal period is considered a critical continuation of the fetal phase that allows the full maturation of the organism. Indeed, key organs and hormonal axes that govern metabolism and energy homeostasis (e.g., hypothalamus, adipose tissue, liver, and gut) undergo structural and functional development after birth. However, species differences exist in pre- and postnatal developmental kinetics. Precocial species, such as non-human primates, give birth to fully mature, vigorous and autonomous infants. Conversely, altricial species, such as rodents, display a shorter gestation time and deliver fragile and immature newborns, usually in litters, which need constant maternal care during their early life [28]. Humans are at the interface between these two definitions and are thus considered secondary altricials. Indeed, while human infants are more developed at birth than other altricial species, the early postnatal period is still of major importance for full organ maturation [28].

The brain is one key organ that undergoes maturation in the early postnatal period. For example, the hypothalamic-pituitary axis, an essential regulator of food intake and energy homeostasis, is relatively immature at birth in both rats and mice. During the first 2 weeks of postnatal life, hypothalamic neurons send axonal projections to their target sites and form functional synapses [29]. Using genome-scale DNA methylation profiling in hypothalamic neurons and non-neuronal cells, Li et al. [30] found that most DNA methylation differences between these two cell types are established postnatally, emphasizing that the early postnatal period is a critical window for cell-type specific epigenetic determination in the murine hypothalamus. In humans, the prefrontal cortex, involved in decision processes such as appetite control and food craving [31], shows an especially prolonged period of postnatal maturation [32,33] that is associated with increased DNA methylation over time [34].

The postnatal period is also a highly sensitive window for white adipose tissue (WAT) development. Although WAT is first detectable at mid-late gestation in humans, the proportion of body fat doubles from ~10% at birth to ~20% by 6 weeks of age [35]. In other species, especially those with a short gestation period, such as rodents, minimal WAT is present prior to birth, and maturation of this tissue occurs primarily postnatally. WAT formation and expansion relies on the determination and differentiation of specific progenitors into adipocytes. Both the adipocyte number and size increase to sustain the major expansion of WAT after birth [36,37]. In mice, subcutaneous inguinal WAT depots start their differentiation around embryonic day 14–18 while visceral gonadal WAT develops exclusively postnatally [38,39,40]. Indeed, Han et al. [39] have shown that the epididymal WAT (eWAT) depot in male mice is generated from a non-adipose structure during the first 14 postnatal days. From postnatal day 1 (P1) to P4, eWAT is composed of multipotent progenitor cells that lack adipogenic differentiation capacity in vitro and can be regarded as being in an ‘undetermined’ state [39]. By P4, progenitor cells from eWAT obtain an adipogenic differentiation capacity [39], suggesting that eWAT undergo drastic epigenetic remodeling in this early postnatal window.

The idea that developmental epigenetics extends beyond embryonic development has been particularly well illustrated in the context of postnatal liver maturation. Indeed, the liver is a key hematopoietic depot during fetal life and acquires its metabolic functions mostly after birth [41]. Recent studies have shown that a majority of the liver-specific methylation pattern is generated postnatally [42,43,44]. Importantly, this observation was similar in purified hepatocytes, indicating that this process is cell autonomous and not the result of changes in cell composition during liver maturation [44]. In particular, genome-wide profiling approaches have revealed that the regulatory regions (e.g., promoters and enhancers) of genes involved in lipid and glucose metabolism undergo programmed active DNA demethylation in a time-dependent manner after birth [44,45,46,47]. This generated specific hepatic unmethylated patterns that were stable, autonomously maintained and required for proper hepatic gene expression through its effects on chromatin accessibility [44].

Interestingly, postnatal active DNA demethylation has been observed in other tissues such as lung, heart, muscle and hippocampus [44,48,49], indicating that many tissues continue to shape their epigenetic landscape after birth. In addition, these data suggest that TETs enzymes, which mediate active DNA demethylation through conversion to 5hmC, could be prime epigenetic actors for postnatal organ maturation. Using a conditional gene ablation strategy in liver, Reizel et al. [44] showed that hepatic active DNA demethylation during the postnatal period is indeed mediated by the timely activity of *Tet2* and *Tet3* enzymes, while *Tet1* is not expressed. Kim et al. [50] recently showed that *Tet1*-deficient mice also display altered postnatal intestinal maturation associated with reduced DNA 5hmC and reduced expression of developmental *Wnt* signaling genes. Conversely, the intestine-specific deletion of *Dnmt1* leads to postnatal death accompanied by impaired postnatal gut maturation [51], while *Dnmt3a* loss was compatible with normal intestinal development. Interestingly, in pancreas, Dhawan et al. [52] showed that during postnatal life, *Dnmt3a* initiates a metabolic program by repressing key genes, thereby enabling insulin secretion in response to glucose levels. Further studies are necessary to elucidate the time- and tissue-dependent roles of TETs and DNMTs isoforms in the postnatal epigenetic maturation of other metabolic organs.

## 3. Early Postnatal Nutrition Affects Offspring Epigenetic Changes

### 3.1. Studies in Humans

While numerous epidemiological studies have linked early infant nutrition in humans (i.e., formula feeding vs. breastfeeding) to adult obesity risk, information about the epigenetic changes associated with this phenomenon is very limited [53]. A longer BF duration is associated with reduced obesity risk in adult life [10] and some studies have assessed the effects of breastfeeding (BF) duration on DNA methylation in infant whole blood [54,55] or buccal epithelial cells [56] (Table 1). These studies reported associations between BF length and DNA methylation near obesity-related genes such as leptin (*LEP*) and retinoid X receptor alpha (*RXRA*) in infants (Table 1). While these three studies suggested a role of BF length in modulating *LEP* promoter methylation, they did not assess the same CpG sites, making conclusions challenging. Moreover, we should emphasize that testing causality between early nutrition and epigenetic programming of obesity in humans is particularly challenging due to the limited access to target tissues that would be relevant for body weight regulation. Nevertheless, in some cases, it has been shown that blood epigenetic markers accurately reflect those of organs such as adipose tissue [57]. Further characterization of whole blood as a proxy measure of metabolic organs’ epigenetic signatures is crucial to facilitate epigenetic programming studies in humans. Finally, other technical considerations such as sample sizes and adjustment for potential confounders (e.g., formula supplementation) should be seriously considered to strengthen the interpretations of association studies in humans.

### 3.2. Studies in Animal Models

Animal models are particularly essential in the study of obesity programming as they allow the analysis of both short and long-term metabolic outcomes in an environmentally controlled setting and provide access to relevant tissues. Several rodent models, such as litter size reduction, artificial enteral high carbohydrate (HC) formula feeding, cross-fostering onto obese mothers, maternal high-fat (HF) feeding exclusively during lactation, and neonatal oral supplementation, have been employed to induce change in breast milk (BM) intake and/or quality and study the consequences of altered nutrition in the lactation-suckling period. Below, we present the characteristics of these animal models and summarize evidence of epigenetic modifications of key metabolic organs in these settings (Table 2).

#### 3.2.1. Litter Size Modulation

Litter size modulation is a well described rodent model which consists of reducing or increasing the number of pups in a litter in order to induce neonatal over- or under-feeding, respectively. Artificially small litters (SL) of three to four pups, versus eight to twelve pups in control litters, allows increased breast milk availability and consumption for SL pups. Studies have shown that milk from SL mothers is also especially enriched in TG compared to control mothers [71,72]. This model induces marked developmental programming of offspring physiology, including hyperphagia, obesity, hyperleptinemia, hyperglycemia and hyperinsulinemia during the early postnatal period, which persists into adulthood [73,74,75].

Using this model, Plagemann et al. [58] first reported that neonatal overfeeding increased hypothalamic DNA methylation in the promoter of the anorexigenic gene proopiomelanocortin (*Pomc*) (Table 2). This hypermethylation was located within two Sp1-related binding sequences that are essential for the mediation of leptin and insulin action on *Pomc* expression. Consequently, SL rats did not display changes in hypothalamic *Pomc* expression, despite hyperleptinemia and hyperinsulinemia, signs of central leptin and insulin resistance. Thus, neonatal overfeeding could program brain satiety pathways via epigenetic modifications. It was recently shown that maternal obesity induced by chronic HF-feeding before mating and throughout gestation and lactation also programs DNA hypermethylation at the *Pomc* promoter in offspring rats [76], suggesting that maternal obesity and neonatal overnutrition could have similar epigenetic programming effects [77]. Li et al. [30] have shown that neonatal overfeeding induced sex-specific changes in DNA methylation of genes involved in hypothalamic neural development (*Aqp14*, *Nolz1*, *Gadd45b*). While these changes translated in gene expression alterations in males, this was not observed in females [30], emphasizing the need to study sex-dependent epigenomic effects.

In a second study, Plagemann et al. [59] reported that neonatal overfeeding increased hypothalamic DNA methylation in the promoter of the insulin receptor (*Insr*) gene, although *Insr* mRNA expression was not changed at weaning (Table 2). Nevertheless, this early epigenetic predisposition could be functionally relevant later in life, participating in the development of hypothalamic insulin resistance that has been described in the SL model [78]. Other groups have observed related epigenetic changes in muscle and liver in association with the development of insulin resistance. For example, Liu et al. [60] reported that SL adult rats presented increased muscle DNA methylation in the promoter of two key insulin signaling genes, insulin receptor substrate 1 (*Irs1*) and the insulin-dependent glucose transporter 4 (*Glut4*). These changes correlated with a decreased expression of both *Irs1* and *Glut4* in muscle [60,79]. While no studies have yet examined WAT epigenome in response to neonatal overnutrition, both *Irs1* and *Glut4* expression levels were also reduced in the epididymal WAT of SL rats [60,79]. Moreover, adipocyte progenitors isolated from subcutaneous WAT of adult SL mice displayed an increased in vitro adipogenic differentiation capacity [80], suggesting that adipocyte precursors could be epigenetically programmed by early overnutrition, similarly to what has been described in the context of maternal obesity [81,82]. In the liver, Ramon-Krauel et al. [61] recently showed that SL offspring displayed an early and persistent increase in monoacylglycerol acyltranferase 1 (*Mogat1*) expression associated with dynamic histone modifications and that elevated *Mogat1* may contribute to adult hepatic insulin resistance. Indeed, Mogat1 converts monoacylglycerol to diacylglycerol (DAG), whose accumulation is known to alter insulin signaling [83]. Importantly, insulin resistance and obesity observed in SL animals can be transmitted to subsequent generations [84,85], reinforcing the role of epigenetic mechanisms in mediating these effects. In addition to insulin resistance, SL offspring also present impaired pancreatic insulin secretion in response to glucose [86]. Using a genome-scale DNA methylation analysis, Li et al. [62] recently observed increased DNA methylation in genes involved in insulin secretion (*Cacna1* and *Scn10a*) both at weaning and in adulthood, suggesting that epigenetic remodeling in pancreatic islets could contribute to the impaired insulin secretion observed in SL animals.

#### 3.2.2. Artificial Rearing Using Enteral Nutrition

Artificial rearing, also known as the “pup in a cup” model, consists of raising newborn rodents in a controlled environment and feeding them an artificial milk formula via an intragastric catheter [87,88,89]. This technique allows complete control over the quality and quantity of nutrition during the suckling period. This model has been extensively used by the group of Patel et al. to study the effects of a high carbohydrate (HC) milk formula (56% kcals from carbohydrates, 20% from fats and 24% from protein) instead of breast milk-like high-fat formula (8% kcals from carbohydrates, 68% fat and 24% proteins). HC milk formula-fed animals develop early and persistent hyperinsulinemia as well as post-weaning hyperphagia, adult onset obesity and insulin resistance [90,91].

At the molecular level, HC animals display expression and epigenetic changes in hypothalamic appetite regulatory genes that could explain the programming of hyperphagia and body weight gain observed in this model. Specifically, HC-reared pups display an increase in hypothalamic expression of orexigenic genes (neuropeptide Y (*Npy*), agouti-gene related polypeptide (*Agrp*)), while the expression of anorexigenic genes (*Pomc*, melanocortin receptor-4 (*Mc4r*), cocaine- and amphetamine-regulated transcript (*Cart*), and corticotrophin-releasing factor (*Crf*)) were decreased [63,92]. These expression changes appeared during suckling and persisted into adulthood [92]. Altered DNA methylation and histone modifications in the *Npy* proximal promoter was observed in the hypothalamus of HC pups and adult animals, while the *Pomc* promoter only showed modification of histone acetylation [63,92] (Table 2). These early and long-lasting alterations suggest that HC formula feeding induces an epigenetic predisposition to hyperphagia. One possible reason for the observed hyperphagia could be related to the source of calories. While the fetus mainly oxidizes glucose, the neonate relies on BM rich in fat with low carbohydrate content. Energy metabolism in the neonate is thus characterized by FA oxidation and ketone body production and utilization [93]. This may be particularly relevant for the postnatal brain which has not yet established efficient glucose-oxidation mechanisms [94]. The combination of neonatal HC feeding and hyperinsulinemia may interfere with hepatic ketogenic activity and could be interpreted by the brain as a sign of energy deficit, thereby promoting epigenetic adaptations to increase food intake and subsequently adiposity.

In addition to obesity, adult HC animals also develop insulin resistance. At the molecular level, Raychaudhuri et al. [64] showed that muscle from HC adult rats display reduced *Glut4* mRNA expression and associated DNA hypermethylation and repressive histone marks in *Glut4* promoter (Table 2). Interestingly, these epigenetic features were associated with the recruitment of DNMTs and histone deacetylases on the *Glut4* promoter [64]. As observed for the SL model, HC female rats transmitted their pathological phenotypes to their offspring which spontaneously developed hyperinsulinemia and adult-onset obesity [95]. Further research using the artificial rearing model will help refine the short- and long-term consequences of specific BM components or formula composition on epigenetic programs in offspring.

#### 3.2.3. Cross-Fostering

The model of cross-fostering is based on the transfer of pups from their nursing mother to a foster mother. This model permits the investigation of the impact of foster mother phenotype, BM quality and behavior on the development of the suckling pups. Several studies have used this model to dissociate the pre- and postnatal effects of maternal diet- or genetically-induced obesity [14,15,96,97]. Pups from lean mothers suckled by obese mothers display increased body weight, adiposity, hyperphagia, hyperinsulinemia and glucose intolerance [14,15,97]. These changes were associated with differences in BM composition, such as increased fat percentage [14] and leptin concentrations [14,97] in obese mothers. Conversely, pups from obese mothers suckled by lean mothers did not display metabolic dysfunctions, indicating that maternal phenotype during lactation-suckling has a dominant influence in determining offspring metabolic phenotype. In agreement, studies in rodents have shown that BM from healthy mothers can override other prenatal susceptibility factors and genetic predisposition to develop obesity [96,98]. While the cross-fostering model has yet to be used to investigate epigenome alterations, these studies again highlight the early postnatal period as a critical window for intervention against obesity risk. However, caution is necessary when interpreting cross-fostering studies. Indeed, it has been shown that cross-fostering alone can program cardiovascular and metabolic dysfunction in adulthood compared to pups nourished by their biological mother [99], reinforcing the need to include proper cross-fostered controls.

#### 3.2.4. Maternal Nutrition Modification Exclusively during Lactation-Suckling

Recent years have seen the appearance of a new model of obesity programming in which lactating mothers are fed a high-fat (HF) diet exclusively during lactation-suckling period [16,66,82,100,101]. Maternal HF feeding during lactation is not accompanied with obesity but was associated with changes in BM composition, such as increases in fat content [65,100], n-6/n-3 PUFA ratio [66] and insulin levels [16,65]. One study reported that HF-fed dams during lactation displayed altered mammary gland structure and function with a slight decrease in pups’ milk yield [101], however, reduced milk intake was not consistently observed [66].

Offspring from HF-fed mothers during lactation displayed increased body weight in early life and adulthood with increased visceral adiposity [16,66,82,101]. Investigating WAT epigenetic programming, work from our group has shown that male offspring rats from HF-fed mothers displayed visceral eWAT expansion associated with increased mRNA and protein expression of the stearoyl-CoA desaturase (*Scd1*) enzyme, a key enzyme of FA metabolism [66] (Table 1). SCD1 converts saturated FAs, e.g., palmitate and stearate, to monounsaturated FAs, palmitoleate and oleate, which are the predominant substrates for TG synthesis. *Scd1* upregulation in eWAT was associated with reduced DNA methylation in *Scd1* promoter surrounding a PPARγ-binding region. Another study showed that offspring from HF-fed mothers display early and persistent altered adaptive thermogenesis [65]. Surprisingly, this was first associated with an increased *Ucp1* mRNA expression in brown adipose tissue (BAT) during suckling as well as increased PPARα binding in *Ucp1* promoter (Table 1). *Ucp1* expression was later decreased in adult HF offspring [82]. However, in-depth epigenetic analysis of *Ucp1* promoter remains to be performed. While neonates from HF-fed dams displayed alterations in lipid and glucose metabolism (e.g., hyperinsulinemia), the persistence of these alterations in adulthood was inconsistent between studies [16,66,82,100]. Further epigenetic examination of this model will likely yield important information about the effects of nutritional intervention in the early postnatal period.

#### 3.2.5. Neonatal Oral Supplementation

Oral supplementation in suckling neonates has been used to assess short- and long-term metabolic outcomes of particular nutritional components. Using this approach, a series of studies has identified leptin as an essential hormone of the lactation-suckling period [102]. Leptin, a hormone produced by WAT, plays a key role in appetite control and metabolism. Leptin is present in BM [103] but not in infant formula [104]. In rodent neonates, orally delivered leptin can be absorbed by the immature stomach, transferred into circulation and regulate food intake during the postnatal period [103,105]. This raised prospects about the potential long-term benefits of BM leptin. Rats orally supplemented with physiological doses of leptin during lactation-suckling were protected against the age-related increase in BW and adiposity and were more resistant to obesity and related complications when exposed to a HF diet in adulthood [68,106,107]. These beneficial effects have been attributed, in part, to improved central and peripheral leptin sensitivity, leading to robust control of food intake and increased peripheral oxidative capacity [108]. Of note, studies delivering postnatal leptin at pharmacological doses and with non-oral administration modes (e.g., subcutaneous injections) have been generally associated with an obese phenotype in adulthood [109,110,111,112], indicating that the dose and mode of administration of postnatal leptin during lactation-suckling are crucial programming parameters.

Oral leptin supplementation during lactation-suckling was also shown to have long lasting effects on the hypothamic expression of factors involved in food intake such as *Pomc*, leptin receptor (*Lepr*) and *Socs3,* an inhibitor of leptin signaling pathway [68]. Interestingly, only *Pomc* expression changes were correlated with changes in DNA promoter methylation in leptin-treated offspring [67] (Table 2). However, these changes in *Pomc* promoter methylation were affected by adult diet [67], suggesting that other mechanisms may participate in the long-term control of food intake by postnatal oral leptin.

Supplementation with other factors during suckling was linked to modification of adiposity in adulthood. Vitamin A supplementation as retinoic acid (RE) during suckling was shown to favor inguinal WAT hyperplasia and body fat gain upon HF diet feeding later in life [70]. Vitamin A supplementation impacted mRNA expression and DNA methylation patterns of genes crucial for WAT development processes, such as adipogenic determination and differentiation (*Zfp423*—hypomethylated, *Pparg*—hypermethylated), cell proliferation (*Pcna*—hypomethylated) but not retinol transport (*Rbp4*—unchanged methylation) in 21 day-old pups [69] (Table 2). These changes suggest that vitamin A supplementation favors the development of iWAT adipocytes with a high proliferative capacity (hyperplasia). Interestingly, gavages of β-carotene, another vitamin A precursor, during the postnatal period, differentially impacts mRNA expression and the DNA methylation patterns of some of these genes (*Pparg*—unchanged methylation, *Pcna*—hypermethylated and *Rbp4*—hypomethylated) at 21 days old [69] (Table 2). However, the resulting metabolic outcomes in offspring were not investigated. Finally, a third study investigated the effects of supplementation with the polyphenol resveratrol and the vitamin B3 form nicotamide riboside during suckling. They found an activated thermogenic/oxidative transcriptional phenotype in iWAT, specifically in male animals, after a 10-weeks HF diet challenge during adulthood [113]. However, the authors did not investigate links between the observed phenotype and epigenetic modulations. These studies highlight the potential of postnatal dietary modification to affect WAT plasticity.

### 3.3. Limitations and Challenges of Epigenetic Studies in Developmental Programming

While much progress has been made in documenting the epigenetic component of obesity programming by early postnatal nutrition, there are still many technical and theoretical questions that merit consideration.

#### 3.3.1. Where, When and How to Look for Epigenetic Reprogramming?

Most of the animal and human studies summarized above reported epigenetic modifications in a single locus, often based on a candidate approach. To assess more globally the impact of early postnatal nutrition on epigenetic programming, it will be crucial to move towards genome-wide screening of methylation using high-throughput technologies. A second major challenge will be the development of data analysis approaches to facilitate the identification of broad epigenetic signatures and interpret their functional consequences in disease development [114]. The biological material in which these studies should be performed is also of critical consideration. Most analysis are performed in whole tissues. However, tissues are composed of many various cell types and there are even examples of heterogeneity among seemingly identical cells (e.g., periportal vs. pericentral hepatocytes, adipocytes from different depots). Consequently, minor epigenetic differences may be due to tissue or cell population heterogeneity and may be a major limitation in these studies. Of note, most epigenetic studies associated with complex diseases in humans and animal models have shown only small changes in methylation (1–10%) [115,116]. It is possible that part of these small variations may be due to variation in cell types within one tissue. The functional importance of relatively modest epigenetic differences and how such variations could contribute to complex phenotypes remains an outstanding question in the field of environmental programming studies [115]. We feel that it is important to consider such changes in the context of complementary measures of gene expression and physiological data. Still, demonstrating that an epigenetic modification induces changes in gene expression and causes an abnormal phenotype remains challenging. The purification of one cell population and/or the recent development and use of single-cell technologies may help overcome this challenge.

#### 3.3.2. Sorting between Correlations and Causality?

By definition, epigenetic marks are heritable, i.e., they remain as the cell divides. However, they are also reversible in response to diverse environmental factors. Thus, the current challenge remains to assess whether these changes are a true cause or simply a consequence of other metabolic disturbances associated with obesity development. One strategy to overcome the influence of external factors on epigenetic marks is to assess whether they persist in vitro in a controlled environment. Many of the studies summarized here have documented epigenetic alterations at only one time point (either in the postnatal period or in adulthood). Investigating the persistence (or not) of pathology-associated epigenetic marks over time is an important step to address the relevance of a given mark. Additionally, the use of technologies designed to specifically manipulate epigenetic marks at genomic loci has opened new avenues to formally link DNA methylation changes to a phenotype. Indeed, modified CRISPR/Cas9 protein in fusion to either DNA methylase (DNMT1) or demethylase (TET1) enzymes allow targeted DNA methylation or demethylation [117] in order to test the direct consequences of these changes. Finally, to establish the line of events that would connect early postnatal nutrition to epigenetic programming of obesity, it is crucial to determine which dietary factors and signaling pathways could drive epigenetic modifications. Below, we explore how BM fatty acids (FA), offspring metabolic and hormonal profiles and gut microbiota may impact epigenetic changes to drive metabolic phenotypes.

## 4. Candidate Pathways Linking Early Postnatal Nutrition and Epigenetic Programming of Obesity?

### 4.1. PPARs Nuclear Receptor as Epigenetic Effectors of Breast Milk Fatty Acids?

BM lipids, especially FAs, are crucial for infant development, providing about 50% of their energy requirement. Interestingly, studies showed that the type of polyunsaturated FAs (PUFAs) in BM influences infant fat deposition and growth. The BM n-6/n-3 PUFA ratio has been positively associated with infant WAT deposition, independently of maternal BMI [11,12]. It is noteworthy that a rise in maternal intake of refined vegetable oils rich in n-6 PUFAs [118] coincides with the significant increase of the n-6/n-3 PUFA ratio in human BM over the last 30 years [118,119]. These results reinforce the previously described obesogenic role of n-6 PUFAs in the perinatal period [118,119,120], and emphasize the importance of BM FA quality as a potential driver of developmental obesity.

FAs can regulate gene expression through modulation of lipid sensing transcription factors [121] and associated DNA methylome changes [122,123]. The peroxisome proliferator-activated receptor (PPAR) family of transcription factors, well known for their FA-activated transcriptional control, have recently been described as key mediators of epigenetic changes. An analysis of mice deficient in PPARα together with maternal administration of a PPARα ligand during the gestation and lactation periods revealed that PPARα activation is required for DNA demethylation of some PPARα target gene after birth [46,47]. These genes include fibroblast growth factor-21 (*Fgf21*), an important hepatokine involved in whole-body energy homeostasis [46,47]. Pharmacological PPARα activation during late gestation and lactation promoted long-term DNA demethylation of *Fgf21* in offspring [47]. This epigenetic memory was associated with enhanced *Fgf21* expression after HF feeding in adulthood and could explain the attenuated HF-diet obesity observed in these animals. In this study, postnatal *Fgf21* DNA demethylation resulted from PPARα-dependent recruitment of the *Tet2* enzyme [47], suggesting that PPARα could act as a scaffold protein to target locus-specific demethylation in liver.

In adipocytes, another study has shown that PPARγ can also induce local DNA demethylation around its binding sites by recruiting TETs enzymes during adipocyte differentiation [124]. Although not in the context of postnatal development, these results suggest that PPARγ could mediate BM FA-induced epigenetic reprogramming in eWAT. Interestingly, we have shown that maternal HF feeding during suckling induces drastic changes in BM FA composition. Moreover, BM FA changes were associated with early and persistent DNA demethylation of the FA desaturase gene *Scd1* at one CpG site located in a PPARγ binding site specifically in eWAT [66]. While these studies suggest that BM FAs could favor PPAR-dependent targeting of TETs for locus-specific active demethylation and epigenetic memory, recent evidence suggests that PPARs could also act via DNMT modulation. PPARγ or PPARα agonist treatments in various cell types has been shown to down-regulate DNMT expression and decrease DNA methylation [125,126]. Finally, PPARγ has been shown to interact with DNMT1 and DNMT3a/b [127,128]. Given the complex interactions between PPARs and the DNA methylation and demethylation machinery, more focused studies are needed to dissect the specific role(s) of these interactions in the context of developmental epigenetic programming.

### 4.2. Impact of Hormonal and Metabolic Imbalance in Neonates?

Interestingly, one common feature of animal models of postnatal programming of obesity is the presence of hyperinsulinemia in the offspring (Table 2). Moreover, BM hormones such as insulin [129] and leptin [103,105] can be transferred to the neonate, where they may be functional [12]. Recent work from Reizel et al. [44] brings strong evidence implicating insulin signaling in postnatal epigenetic reprogramming of liver. Using a mouse model of conditional *Insr* deletion in liver during the postnatal period, the authors showed that *Insr* deletion after birth abolished the demethylation of approximately 40% of regions that were otherwise shown to undergo physiological demethylation during liver maturation [44]. Moreover, some insulin target genes (*Glut4*, *Scd1, Irs1* etc.) have been shown to be affected in animal models of postnatal programming (Table 1). Finally, there is a well described gender effect of metabolic programming [130] that may be a result of differences in sex hormones, such as testosterone. Indeed, testosterone secretion triggers DNA methylation changes that occur specifically in the male liver [42]. Further studies are needed to delineate the role of neonatal hormonal milieu in epigenetic reprogramming.

Hyperglycemia, also a frequent feature of postnatal obesity programming models, may also play a direct role in epigenetic modification. For example, TET enzymes are known to interact with O-GlcNAc transferase (OGT), which is activated by hyperglycemia to promote OGT binding to DNA thereby inducing histone modifications [131]. Interestingly, a recent study has shown, in HepG2 hepatoma cells, that increased OGT activity was associated with increased TET activity and led to a hyperhydroxymethylation at some gene promoters, such as SREBP-1c [132], which would favor lipogenic activity. Thus, we propose that the deregulation of glucose homeostasis during postnatal development could partially drive epigenetic reprogramming and disease susceptibility.

### 4.3. A Role for Gut Microbiota?

BM composition is complex and includes macronutrients, micronutrients and a wide range of non-nutritive bioactive factors such as hormones, growth factors, microRNAs, cells as well as prebiotics. All of these could impact infant development. Recently, a higher total concentration of human milk oligosaccharides (HMO) has been shown to be negatively associated with infant adiposity [133,134], suggesting that HMO could participate in obesity protection linked to breastfeeding. HMO are complex glycans that are highly abundant in human milk but not infant formula, and may act as prebiotics. Thus, HMO could protect from fat accretion by promoting healthy microbiota and microbiota-derived metabolites like short chain fatty acids (SCFAs) [135,136]. Interestingly, a recent study has shown that the SCFAs butyric and formic acid are also present in BM, and associate negatively with infant BMI [137]. Moreover, considering that metabolites from some gut bacteria can act as substrates or modulators of DNA methylation processes [138], it is possible that epigenetic effects of early postnatal nutrition originate from modification of gut microbiota during the postnatal period. Interestingly, a recent study showed that the gut microbiota is able to drive methylome changes in intestinal epithelial cell during postnatal development [139]. Further studies are needed to explore the exciting relationship between postnatal nutrition, infant gut microbiota and epigenetic programming.

## 5. Conclusions

**Figure 2:**
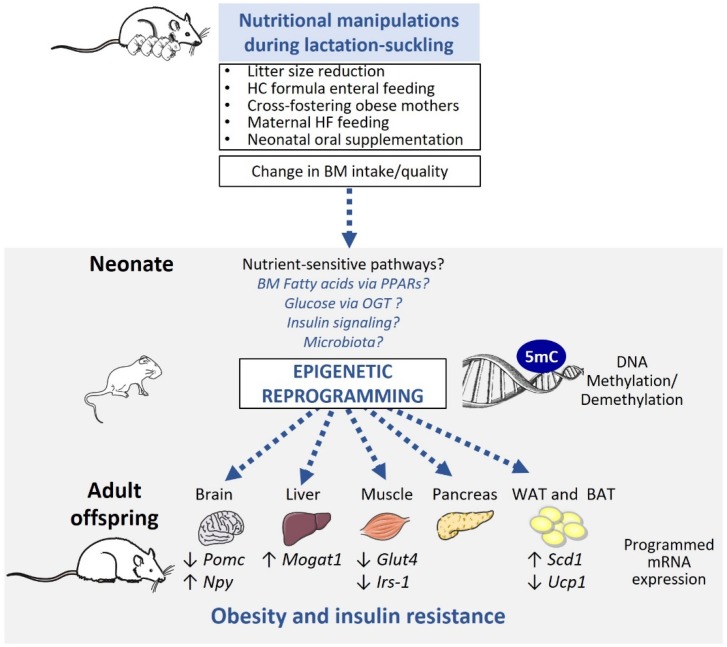
Animal models of postnatal programming of obesity include litter size reduction, artificial enteral high carbohydrate (HC) formula feeding, cross-fostering onto obese mothers, maternal high-fat (HF) feeding during lactation or neonatal oral supplementation. These models lead to direct or indirect change in neonatal breast milk (BM) intake and/or quality (nutrients, hormones, etc.). The ensemble of these interventions have been shown to induce targeted epigenetic modifications (e.g., DNA methylation or demethylation, and histone modifications) associated with decreased (↓) or increased (↑) mRNA gene expression in key metabolic organs such as brain, liver, muscle, pancreas, white and brown adipose tissue (WAT and BAT respectively)) (see Section 3.2). While the molecular mechanisms underlying these effects are largely unknown, we discuss the potential epigenetic role of BM fatty acids (FAs) and PPARs nuclear receptors, neonatal metabolic and hormonal milieu (glucose/insulin) and gut microbiota.

A wealth of evidence now indicates that early postnatal nutrition, independently of in utero exposure, can program lifelong obesity risk. As summarized in Figure 2, evidence mostly from animal models shows that changes in early postnatal nutrition quality and/or intake influence offspring epigenetic and expression profile in various organs involved in body weight regulation. However, the idea that nutrition-induced epigenetic programming is a true cause of obesity development is still very limited. Additional studies are urgently needed to clarify the physiological relevance of epigenetic dysregulation in disease programming. While it is difficult to prove causality in human studies, the analysis of samples before and after exposure (e.g., at birth and later in life) is essential to dissect the precise consequences of environmental changes. As a complementary approach, animal studies are fundamental, since they provide, among other possibilities, access to tissue/cells, which can be studied ex vivo to test the nature and properties (persistence, reversibility etc.) of epigenetic marks identified in vivo. We also believe that a better molecular understanding of the epigenetic-modifying properties of nutritional and metabolic factors is imperative to address whether epigenetic modifications are simply correlative or true drivers of obesity development. In this context, we believe it is crucial to explore the complexity of BM composition and its relationship to infant fat accretion. While BM is seen as the gold standard for infant nutritional requirements and for its long-term health benefit, recent evidence indicates that BM’s properties may be modulated by its composition. Identifying factors (e.g., maternal, environmental) that influence BM composition could help dissect potential confounders of obesity programming. Altogether, these efforts should help refine the actors involved in the developmental programming of obesity, and allow the development of epigenetic biomarkers or epigenome-targeted interventions to combat obesity.

## Figures and Tables

**Table 1:** Associations between breastfeeding length and gene promoter methylation in humans. For each study, details on cohort (number, age, groups), sample type and analysis (gene, number of CpGs, detection methods) as well as findings are indicated.

Study	Cohort	Sample Type and Analysis	Findings
Obermann-Borst et al., 2013 [54]	98 infants at 1.4 years-old; BF duration groups (number/group): No BF (24), <1 month (14), >1–3 months (21), >3–6 months (21), >6 months (18)	Whole blood 10 CpGs of *LEP* promoter; Mass spectrometry-based method with bisulfite DNA conversion	Longer BF duration ↓ mean CpG methylation of *LEP* *LEP* CpG methylation is negatively associated with plasma leptin and infant BMI
Pauwels et al., 2019 [56]	101 infants at 1 year-old (42.5% girls); BF duration groups (number/group): No BF (5), 1–3 months (31), 4–6 months (29), 7–9 months (19), 10–12 months (17)	Buccal epithelial cells; 1 CpG *LEP* promoter 5 CpG *RXRA* promoter; Pyrosequencing	↑ CpG3 methylation *LEP* promoter if only BF length = 7–9 months Longer BF duration ↑ CpG2 and ↓ CpG3 methylation of *RXRA*
Sherwood et al., 2019 [55]	259 infants at 10 years-old; 257 infants at 18 years-old Groups (number/group not available): exclusive BF vs. mixed feeding (BF despite formula feeding or solid food introduction)	Whole blood 16 to 23 CpGs of *LEP*; Infinium methylation EPIC BeadChips or Infinium Human Methylation450	10 year-old infants: exclusive BF is associated with increased or decreased methylation of various CpGs in *LEP* promoter One CpG (cg23381058) methylation status is positively associated with a BMI trajectory toward an early transient obesity in both total and exclusive BF No associations in 18 year-old infants

BF, breastfeeding; BMI, body mass index; CpG, Cytosine-Guanine dinucleotide; *LEP*, leptin; *RXRA*, retinoid X receptor alpha.

**Table 2:** Summary of epigenetic modifications reported in animal models of obesity programming induced by altered nutrition in the lactation-suckling period. For each study, we indicated animal species, sex (F, female; M, male), pups per litter, diet characteristics, and age-related metabolic and molecular outcomes (epigenetic modulation and gene expression) in the offspring.

Model	Study Details	Metabolic Outcomes	Epigenetic Modifications Gene Expression
**Litter Size Adjustment**			
Plagemann et al., 2009 [58]; Plagemann et al., 2010 [59]	Rat 12 vs. 3 pups per litter	At 21 days old: ↑ BW, adiposity ↑ Plasma glucose, insulin, leptinInsulin resistance	Hypothalamus *Pomc*: ↑methylation at CpGs 12–13 (Sp1 and NF-Kb binding sites), but no change in mRNA *Npy*: ↔ methylation at CpGs 1–17 *InsR*: ↑ mean CpGs methylation in the −322bp upstream CGI of InsR promoter, but no change in mRNA
Liu et al., 2013 [60]	Rat (F) 12 vs. 3 pups per litter	At 21 days old: ↑ BW ↑ Plasma insulin	Muscle *Irs1:* ↔ methylation at CpGs 4–21 ↓ mRNA *Glut4*: ↓ methylation at CpG 5 ↑ mRNA
		At 4.5 months old: ↑ BW, food intake ↑ Plasma glucose, insulin, leptin	Muscle *Irs1*: ↑ methylation at CpGs 8, 9–12, 15–17 ↓ mRNA *Glut4*: ↑ methylation at CpGs 13–14 ↓ mRNA
Ramon-Krauel et al., 2018 [61]	Mouse (M) 8 vs. 4 pups per litter	At 14 days old: ↑ BW, food intake ↑ eWAT ↔ Plasma glucose, insulin, TGs; ↑ Plasma NEFA	Liver *Mogat1*: ↔ methylation at CpGs 1–21 ↓ mRNA
		At 6 months old: (At 4 months-old): ↑ Plasma insulin, TGs, liver TGs content; ↔ Plasma glucose, NEFA	Liver *Mogat1*: ↔ methylation at CpGs 1–21 ↑ enrichment H3K4me3, H3K9ac ↑ mRNA
Li et al., 2013 [30] Li et al., 2019 [62]	Mouse (F & M) 9 vs. 4 pups per litter	At 21–25 days old: ↑ BW, adiposity (F & M)	Hypothalamus *Aqp14*: ↑ methylation (*p* = 0.06, F only), but no change in mRNA *Nolz1*: ↑ methylation (*p* = 0.07, F only), but no change in mRNA *Gadd45b*: ↑ methylation (M only) ↓ mRNA Pancreas (only M studied): ↑ global genomic methylation *Akt1*: ↑ methylation *Cacna1i*: ↑ methylation *Scn10a*: ↑ methylation
		At 6 months old: ↑ BW, adiposity (F & M) ↓ Energy expenditure (F only)	Hypothalamus *Aqp14*: ↑ methylation (*p* = 0.06, F only), but no change in mRNA *Nolz1:* ↑ methylation (*p* = 0.07, F only), but no change in mRNA *Gadd45b*: ↑ methylation (M only) ↓ mRNA Pancreas (only males studied) ↑ global genomic methylation *Akt1*: ↔ methylation *Cacna1i*: ↑ methylation *Scn10a*: ↑ methylation
**Artificial Rearing**			
Mahmood et al., 2013 [63]	Rat (F) HC artificial formula (56% carbohydrate, 20% fat, 24% protein in kcal) vs. maternal rearing (maternal milk: 8% carbohydrate, 68% fat, 24% protein in kcal)	At 16 days old: ↔ BW ↑ Plasma insulin ↓ Plasma leptin	Hypothalamus *Pomc*: ↔ methylation at CpGs 2–23 ↓ H3K9ac enrichment ↔ H3K9me2 enrichment ↓ mRNA *Npy*: ↑ methylation at CpG21 ↓ mean methylation at CpGs 1–24 (*p* = 0.06) ↑ H3K9ac enrichment ↔ H3K9me2 enrichment ↑ mRNA
		At 3 months old: ↑ BW ↑ Plasma insulin, leptin	Hypothalamus *Pomc*: ↔ methylation CpGs 2–23 ↔ mRNA *Npy*: ↑ methylation CpG 21 ↓ methylation CpGs 1–2, 16–17, 20, 24 ↑ mRNA
Raychaudhuri et al., 2014 [64]	Rat (M) HC artificial formula (56% carbohydrate, 20% fat, 24% protein in kcal) vs. maternal rearing (maternal milk: 8% carbohydrate, 68% fat, 24% protein in kcal)	At 12 days old: ↓ Plasma TSH, T4	Muscle *Glut4*: ↔ mRNA
		At 3 months old: ↓ Plasma TSH	Muscle *Glut4*: ↑ mean CpG methylation ↑ DNMT3b (CpG1-2), DNMT3a (CpG3) binding ↓ TR, SRC-1 and CBP binding ↑ MeCP2, HDAC4 binding ↓ H4K16ac enrichment ↓ mRNA
**Maternal Nutrition**			
Liang et al., 2016 [65]	Mouse (M) Maternal HF (60% kcal fat) vs. C (10% kcal fat) diet6 pups per litter	At 21 days old: ↑ BW ↑ eWAT, iWAT, BAT ↑ Plasma glucose, TGs	BAT *Ucp1*: ↑ PPARa binding (ChIP) ↑ mRNA
		At 4 months old: ↑ BW ↑ eWAT, iWAT, BAT Insulin resistance, glucose intolerance	BAT *Ucp1*: ↓ mRNA
Butruille et al., 2019 [66]	Rat (M) Maternal HF (60% kcal fat) vs. C (10% kcal fat) diet 8 pups per litter	At 12 days-old: ↑ BW, eWAT, iWAT (hypertrophy) ↔ Plasma glucose, TGs ↑ Plasma leptin, insulin (*p* = 0.08)	eWAT *Scd1*: ↓ methylation CpG33 (*p* = 0.08) ↓ mRNA
		At 6 month-old: ↑ BW, eWAT (hyperplasia) ↔ iWAT ↔ Plasma glucose, insulin, leptin ↑ Plasma adiponectin (*p* = 0.08)	eWAT *Scd1*: ↓ methylation CpG33 ↑ PPARg binding (ChIP) ↑ mRNA
**Neonatal Supplementation**			
Palou et al., 2011 [67] Picó et al., 2007 [68]	Rat (M) Daily leptin gavage (5 fold BM physiological dose) vs. C gavage (water) from postnatal day 1 to 20	At 6 months old (adult C or HF diet): ↓ BW, food intake (postnatal leptin protective effect against age-induced and HF-induced obesity) ↔ Plasma glucose, insulin, leptin, ghrelin	Hypothalamus *Pomc*: ↓ methylation CpG6 ↓ mRNA But for offspring exposed to HF during adulthood: ↑ methylation CpG6 ↑ mRNA *LepR*: ↔ methylation CpGs 1–19 ↔ mRNA *Socs3*: ↔ methylation CpGs 1–13 ↔ mRNA
Arreguín et al., 2018 [69] Granados et al., 2012 [70]	Rat (M) Daily retinyl ester gavage (3–5 fold BM physiological dose) vs. C gavage (olive oil) from postnatal day 1 to 20	At 21 days old: ↔ BW, fat mass ↑ iWAT proportions of small adipocytes (At 4.5 months old: ↑ iWAT, eWAT mass following HF diet during adulthood)	iWAT *Pparg2*: ↑ methylation CpGs 1–4 ↓ mRNA *Rbp4*: ↔ methylation CpGs 1–9/13–37 ↓ mRNA *Zfp423*: ↓ methylation CpGs 1–3 ↑ mRNA *Pcna*: ↓ methylation CpGs 1–17 ↔ mRNA
	Rat (M) Daily β-carotene gavage (3–5 fold BM physiological dose) vs. C gavage (olive oil) from postnatal day 1 to 20	At 21 days old: (NA)	iWAT *Pparg2*: ↔ methylation CpGs 1–4 ↔ mRNA *Rbp4*: ↓ methylation CpGs 1–9/13–37 ↔ mRNA *Zfp423*: ↓ methylation CpGs 1–3 ↑ mRNA *Pcna*: ↑ methylation CpGs 1–17 ↔ mRNA

*Akt1*, Protein kinase B; Aqp14, aquaporin 14; BAT, brown adipose tissue; BM, breast milk; bp, base pairs; BW, body weight; C, control; *Cacna1i*, Calcium Voltage-Gated Channel Subunit Alpha1 I; CBP, CREB-binding protein; CGI, ChIP, chromatin immunoprecipitation; CpG island; CpG, Cytosine-Guanine dinucleotide; DNMT, DNA methyl transferase; eWAT, epididymal white adipose tissue; F, females; FAs, fatty acids; *Gadd45b*, Growth arrest and DNA-damage-inducible b; *Glut4*, glucose transporter 4; HC, high carbohydrate; HDAC4, histone deacetylase 4; HF, high fat; *InsR*, insulin receptor; *Irs1*, Insulin receptor substrate 1; iWAT, inguinal white adipose tissue; *LepR*, leptin receptor; M, males; MeCP2, methyl CpG binding protein 2; *Mogat1*, monoacylglycerol O-acyltransferase 1; mRNA, messenger RNA; NA, not available; NF-Kb, nuclear factor kappa-B; NEFA, non-esterified fatty acids; *Nolz1*, Zinc Finger Protein 503; *Npy*, neuropeptide Y; *Pcna*, proliferating cell nuclear antigen; *Pomc*, pro-opiomelanocortin; *Pppar*, peroxisome proliferator-activated receptor; *Rbp4*, retinol binding protein 4; *Scd1*, stearoyl-coA desaturase 1; *Scn10a*, Sodium Voltage-Gated Channel Alpha Subunit 10a; *Socs3*, suppressor of cytokine signaling 3; Sp1, specificity protein 1; SRC1, steroid co-activator 1; T4, thyroxine; TGs, triglycerides; TR, thyroid hormone receptor; TSH, thyroid-stimulating hormone; *Ucp1*, uncoupling protein 1; *Zfp423*, zinc finger protein 423.
